# Supplementary material for: Targeting Mitochondria in Melanoma
Source: Biomolecules. 2020 Sep 30;10(10):1395. doi: 10.3390/biom10101395 (PMC7599575; doi:10.3390/biom10101395)
Supplement: Supplementary file 1 [file biomolecules-10-01395-s001.pdf]

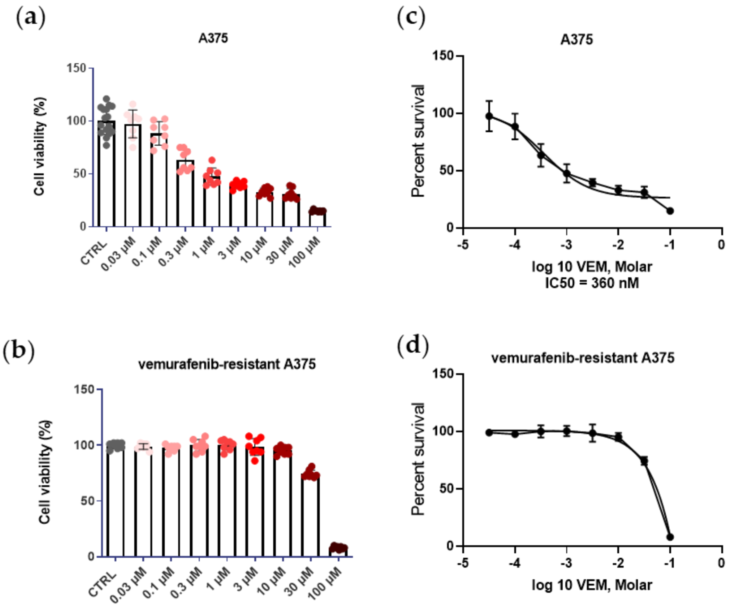

Supplementary Figure S1. Effect of vemurafenib on the growth of monolayer cultures of RA375 and A375 cells at the indicated concentrations for 72 hours (**a**). (**b**) Dose–response curve to generate IC50 of vemurafenib in the RA375 and A375 cell lines.

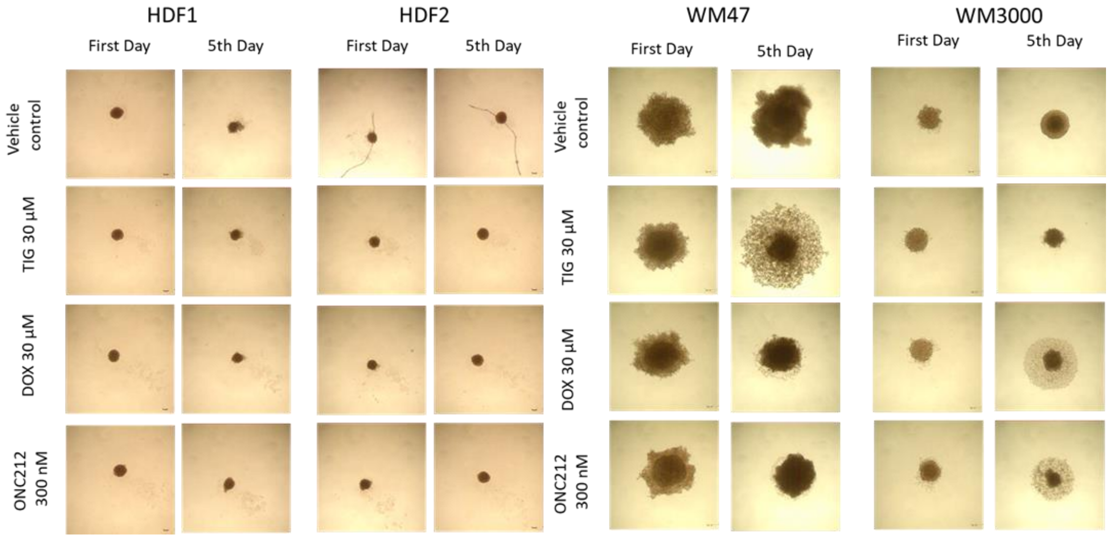

Supplementary Figure S2. Volume of HDFs and melanoma spheroids after treatment with TIG, DOX (30 µM) and ONC212 (300 nM). Images were acquired at day 1 and day 5 of treatment. Photos were taken at 100× magnification.
